# CoviWall, a whole-virion-inactivated B.1.617.2 vaccine candidate, induces potent humoral and Th1 cell response in mice and protects against B.1.617.2 strain challenge in Syrian hamsters

**DOI:** 10.3389/fimmu.2024.1447962

**Published:** 2025-01-22

**Authors:** Jyotsna Dandotiya, Neeta Adhikari, Manas Ranjan Tripathy, Kamini Jakhar, Sudipta Sonar, Dibya Ranjan Pati, Vibhu Kanchan, Varsha S. Prasad, Jitendra Kumar, Nitesh K. Senapati, Arti Bharmoria, Neeraj Rani, Monika Lakhanpal, CS. Patil, Nishan Singh, Lovely Khan, Lavit Jambu, Naveen K. Jain, Syed Khalid Ali, Priyanka Priyadarsiny, Amulya K. Panda, Rajesh Jain, Shailendra Mani, Sweety Samal, Amit Awasthi, Zaigham Abbas Rizvi

**Affiliations:** ^1^ Immuno-biology Lab, Centre for Immunobiology and Immunotherapy, Translational Health Science and Technology Institute, Near Capital Region (NCR)-Biotech Science Cluster, Faridabad, Haryana, India; ^2^ Immunology-Core Lab, Translational Health Science and Technology Institute, Near Capital Region (NCR)-Biotech Science Cluster, Faridabad, Haryana, India; ^3^ OneStream Research Centre, Panacea Biotec Limited, New Delhi, India; ^4^ Translational Health Science & Technology Institute (THSTI), NCR-Biotech Science Cluster, Faridabad, Haryana, India

**Keywords:** CoviWall, COVID-19-vaccine, immunogenicity, hamster, Delta strain, Th1, humoral response

## Abstract

Rapid development of coronavirus disease 2019 (COVID-19) vaccines and antiviral drugs have significantly reduced morbidity and mortality worldwide. Although most of the vaccines were developed initially with the ancestral Wuhan antigen, here, we report the development and immunological efficacy of a whole-virion–inactivated vaccine candidate (CoviWall) to combat the deadly B.1.617.2 (Delta strain) infection. In the current study, we demonstrate a consistent manufacturing process under Good Manufacturing Practice for the development of CoviWall and its characterization using various analytical methods as per regulatory compliance. In addition, we provide pre-clinical immunogenicity and protective efficacy data of the CoviWall vaccine. All the three test doses (i.e., low dose, mid dose, and high dose) immunized in C57BL/6 mice elicited a high titer of anti–receptor-binding domain antibody and neutralizing antibody response against severe acute respiratory syndrome coronavirus–2 (SARS-CoV-2) after second booster dose. In addition, CoviWall immunization also produced a significant T-cell response in the immunized animals. Our B.1.617.2 strain challenge data in Syrian hamsters indicate that immunized hamsters show attenuated clinical manifestations of COVID-19 with reduced lung viral load. Moreover, assessment of pulmonary histopathology revealed lower cellular injury, inflammation, and pneumonia in the vaccinated hamsters as compared to the unvaccinated animals. Such promising results augur well for the clinical phase I trial of the CoviWall vaccine and further development against contagious SARS-CoV-2 strains in the future.

## Introduction

Coronavirus disease 2019 (COVID-19) has caused a huge global healthcare crisis with tremendous loss of human life and the economy. Based on the reported cases, over around 10% of the world population has encountered active severe acute respiratory syndrome coronavirus–2 (SARS-CoV-2) infection at least once with close to 1% mortality (https://data.who.int/dashboards/covid19/cases?n=c). SARS-CoV-2 belongs to the family *Coronaviridae* and contains positive-sense single-stranded RNA as the genetic material that encodes the structural proteins (S), envelope (E), membrane (m), and nucleocapsid (N), out of which spike protein is major protective antigen (1, 2). Spike protein assists in angiotensin-2–converting enzyme (ACE2)–mediated host cellular entry and gets cleaved into S1 and S2 fragments by transmembrane serine protease-2 (3). The receptor-binding domain (RBD), part of the S1 fragment, interacts with the ACE2 receptor and is crucial for viral entry.

Acquired mutations in the genome of SARS-CoV-2 have influenced the virus pathogenesis and infectivity. Apart from the previous variants of concern (VoCs) of SARS-CoV-2, namely, Alpha (B.1.1.7), Beta (B.1.351), Gamma (P.1), Delta (B.1.617.2), Epsilon (B.1.427 or B.1.429), and Omicron (B.1.1.529), new VoCs like BA.5 and JN.1 have emerged recently, which has led to global surge and new waves of COVID-19 (4–6). Among these, the Delta (B.1.617.2) strain caused one of the deadliest COVID-19 waves in India and across the globe (4, 7). The Delta variant was first detected in India in December 2020, which then quickly spread across the globe with the first reported case in the USA in April 2021. Remarkably, the B.1.617.2 variant harbors as many as 10 mutations [T19R, (G142D*), 156del, 157del, R158G, L452R, T478K, D614G, P681R, and D950N] in the spike protein (4, 8). This constant evolution of the virus has led to new waves of COVID-19 with increased cases of hospitalization and mortality. Moreover, it was also found that vaccine candidates developed on the backbone of ancestral Wuhan strain were lesser effective against emerging VoCs with poorer humoral response (9, 10). Several vaccine platforms have been described in the literature, which include live-attenuated vaccines, inactivated vaccines, subunit vaccines, viral vector vaccines, nucleic acid vaccines (DNA/RNA), and protein-based vaccines, each with unique features. Live-attenuated vaccines, like Measles, Mumps, and Rubella (MMR) Vaccine, mimic natural infection, eliciting strong immunity but carrying the risk of reversion to virulence in immunocompromised individuals. Inactivated vaccines, like polio vaccines, are safer but may induce weaker immunity, requiring adjuvants or boosters. Subunit vaccines, like Hepatitis B, are highly specific and safe but often less immunogenic and need adjuvants. Viral vector vaccines, such as those for COVID-19, deliver strong immunity but face concerns about pre-existing vector immunity. Nucleic acid vaccines (e.g., mRNA-based) offer rapid production and strong cellular immunity but are limited by cold-chain requirements and relatively novel safety profiles. Protein-based vaccines are safe and well tolerated but may require complex manufacturing and adjuvant support. Each technology represents trade-offs between efficacy, safety, and logistical feasibility, tailored to specific diseases and population needs (11). However, in the early COVID-19 pandemic stage, only three vaccine candidates have received Emergency Use Authorization: Pfizer/BioNTech and Moderna (mRNA vaccine), Johnson & Johnson (viral vector vaccine) in the US, and the University of Oxford/AstraZeneca (viral vector vaccine) in the UK (12). During the early phase of COVID-19, India has only two vaccine options available: 1) an adenovirus-based vaccine using ancestral spike protein as antigen and 2) whole-virion–inactivated vaccine that uses Indian isolate of the ancestral strain of SARS-CoV2 (13, 14). During the time when the entire world was grappling with the deadly Delta surge, vaccines based on ancestral viruses were being administered to the population. However, these vaccines were less effective and showed a poor humoral response (9). This made it challenging to develop a vaccine specifically targeting the Delta strain within a short timeframe. However, since India had already created an inactivated virion–based vaccine for the ancestral strain, it was deemed sensible to quickly adapt this same and safe strategy to develop an inactivated virion–based vaccine for the Delta variant. In line with this, we utilized an isolated Delta strain that was circulating in India to develop the CoviWall, a whole-virion–inactivated vaccine candidate. CoviWall, a vaccine different from mRNA and viral vector vaccines, has the potential to induce a robust immune response to multiple viral antigens not just spike proteins. This mammalian cell culture–based vaccine does not require ultra-cold storage like the mRNA vaccine, making it easier to distribute in resource-limited settings. This vaccine uses a safe and well-established platform, which has been previously characterized and proven to be safe. This study utilized that established well-characterized platform for the development of CoviWall (15–17). For this purpose, a GMP process was used to produce and purify inactivated antigens and characterize them further to ensure the standard quality. After qualifying the initial QC steps, additional biophysical characterization and *in vitro* evaluation were performed. The study assessed the immunogenicity and protective efficacy of the vaccine using a mouse and hamster model. A mouse model demonstrates the primary immune response to vaccines, whereas a hamster model mimics human respiratory diseases, such as COVID-19, aiding in understanding respiratory virus pathogenicity and the impacts of vaccines efficacy and safety. The C57BL/6 mice immunized with two booster doses of CoviWall produced a significant humoral response with high antibody titers against RBD and neutralizing antibodies when compared to the unimmunized mice. Furthermore, Th1-skewed response with upregulated Interferon-gamma (IFN-γ) level was seen in immunized mice as compared to that in unimmunized mice, indicating a positive correlate of protection as shown previously. Next, we used the Syrian hamster model for a protective efficacy study. CoviWall immunization rendered protection against the B.1.617.2 (Delta) strain–with reduced clinical pathology, significantly mitigated lung viral load and body mass loss [mid dose (MD) and high dose (HD)]. Furthermore, histological assessment of pulmonary pathology revealed an overall decrease in pathological scores and disease index in immunized hamsters, as compared to unimmunized hamsters. In addition, significant levels of serum-neutralizing antibodies were also generated in the CoviWall-immunized animals. Taken together, our pre-clinical animal data show that CoviWall immunization produces an efficient protection against B.1.617.2 strain infection, which is based on strong humoral and Th1 cell–based immune response. This study could form the basis for future assessment of CoviWall in clinical trials.

## Results

### Batch manufacturing and characterization of whole-virion–inactivated SARS-CoV-2 Delta variant vaccine

The continuous mammalian cell line used for manufacturing (Vero-based) was tested and found to comply with its growth characteristics and morphology: sterility and viability both at the Master Cell Bank (MCB) level and Working Cell Bank (WCB) level. The MCB and WCB were also tested for the presence of adventitious agents (DNA/RNA viruses) and found to be free from any/all such adventitious agents. Three consistency reproducible batches (DLT 2104, DLT 2105, and DLT 2106) were generated using the optimized manufacturing process following current Good Manufacturing Practice (cGMP) guidelines, where the upstream processing titer of virus harvest was maintained not less than 7 × 10^6^/mL (TCID_50_), with a scalable volume of 5 L per batch. The virus inactivation by Beta-propiolactone (BPL) was confirmed by testing for the presence of any residual live virus test after inactivation as per in-house protocol no. ARD/D/CoV/002. The results were evaluated for all the different time-point samples in six-well plates with four replicates per sample. No cytopathic effect (CPE) was observed in any of the test wells, similar to the negative control. It was concluded that the virus bulk after inactivation was free from potent live virus at all time points.

The virus bulk also retained the antigenic integrity of the structural protein as analyzed by Enzyme-linked immunosorbent assay (ELISA) (18). After inactivation, the drug substance was purified following the established purification strategy, and analytical characterization was carried out for its purity and potency. The result indicated the drug substance as a clear homogeneous liquid, free from any extraneous particles and any CPE, suggesting complete virus inactivation. The drug substance was identified as SARS-CoV-2 by detection of the specific spike protein by ELISA. It was also found to have a purity of >95% as assessed by sodium dodecyl sulfate-polyacrylamide gel electrophoresis (SDS-PAGE). Apart from this, the drug substance was found to be free from any residual inactivating agent (BPL), residual trypsin, residual endonuclease, and bioburden. **Table 1** provides the key highlights of the process parameters of the entire manufacturing process (upstream, downstream, and analytical) for the three consistency batches of the drug substance (DLT 2104, DLT 2105, and DLT 2106) manufactured under cGMP at Panacea Biotec Ltd., New Delhi.

**Table 1:** Process parameters of the CoviWall manufacturing process.

Stage	Process parameter	Results		
		DLT 2014	DLT 2105	DLT 2106
Upstream processing	Virus harvest titer (TCID_50_)	7 × 10^6^/mL	1.56 × 10^7/^mL	2.43 × 10^7/^mL
	Virus harvest volume	5,006 mL	5,006 mL	5,007 mL
Downstream processing	Drug substance bulk was processed and sterile-filtered.	Qualifies	Qualifies	Qualifies
Analytical testing of DS	pH (Potentiometric)	7.49	7.32	7.28
	Potency (total protein content by micro-BCA assay)	56.48 µg/mL	55.41 µg/mL	54.55 µg/mL
	Endotoxin content (gel-clot method; semi-quantitative)	< 50 EU/dose	< 50 EU/dose	< 50 EU/dose
	Residual BSA content (ELISA)	37.09 ng/dose	40.96 ng/dose	41.50 ng/dose
	Residual host cell DNA content (qPCR)	0.003 ng/dose	0.012 ng/dose	Absent

(1 Dose = 6 µg of total protein).

**Figure 1:**
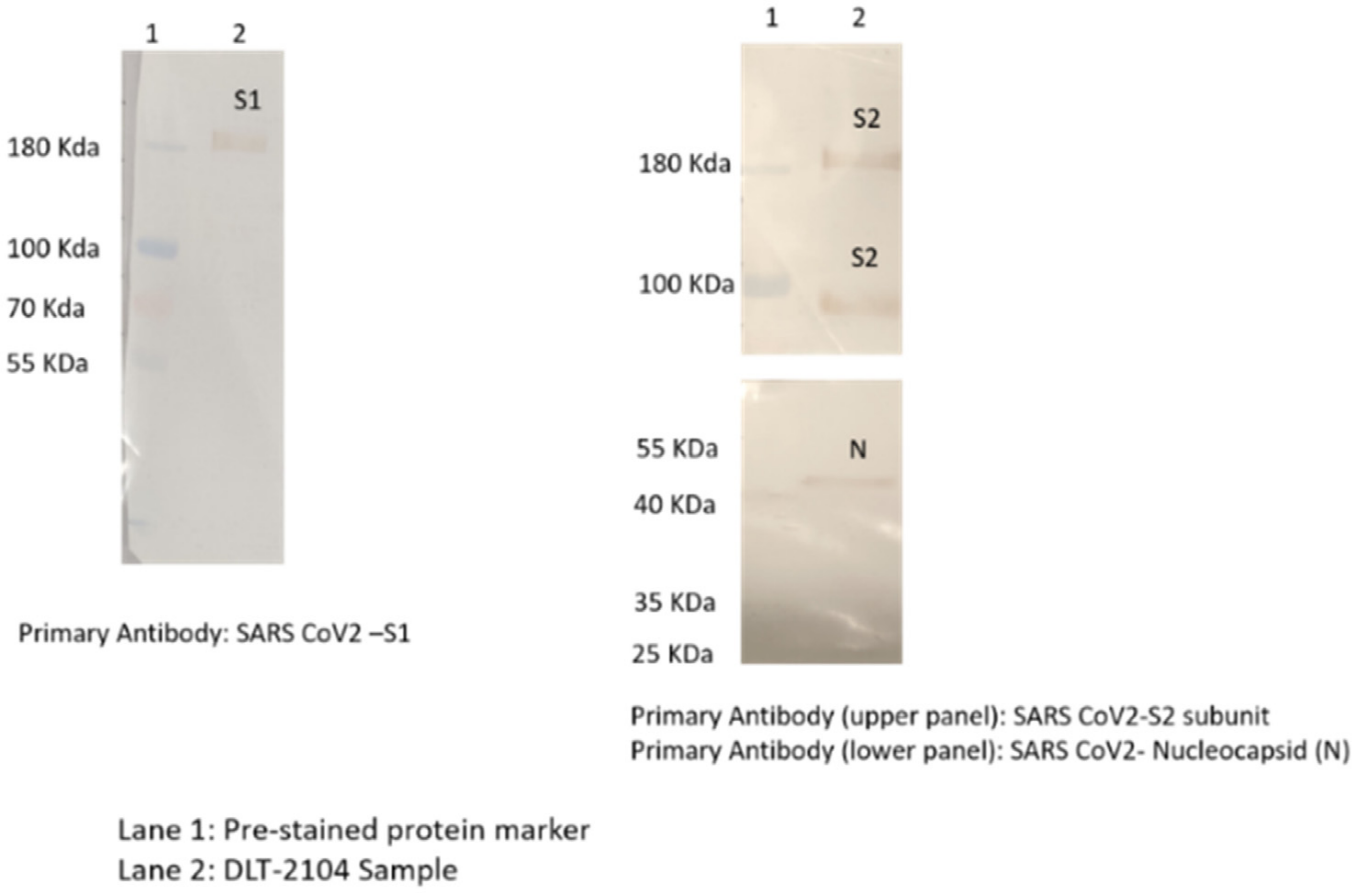
Western blotting for analyzing specific proteins of SARS-CoV-2. Immunoreaction was observed as follows: (1) above 180-kDa region with SARS-CoV-2 S1 primary antibody; (2) above 180-kDa (for uncleaved) and near 100-kDa region (for cleaved and dissociated) with SARS-CoV-2 S2 primary antibody; and (3) near 55-kDa region with SARS-CoV-2 nucleocapsid (N) primary antibody.

The drug substances of all three consistency batches qualified the acceptance criteria as per the drug substance release specifications. Additionally, the following biological and physiochemical characterization of the drug substance from the three consistency batches were also performed:

The epitope integrity of the virus (SARS-CoV-2) after BPL inactivation was confirmed by Western Blotting with the following details:

Specific immuno-reactions corresponding to the analyzed structural proteins like S1, S2, and N of the SARS-CoV-2 virus were observed (**Figure 1**). Specifically:

1. Immunoreaction above 180-kDa region with SARS-CoV-2 virus S1 primary antibody was observed.
2. Immunoreaction above 180-kDa (for uncleaved) and/or near 100-kDa region (for cleaved and dissociated) with SARS-CoV-2 virus S2 primary antibody was observed.
3. Immunoreaction near 55-kDa region with SARS-CoV-2 N primary antibody was observed.

**Figure 2:**
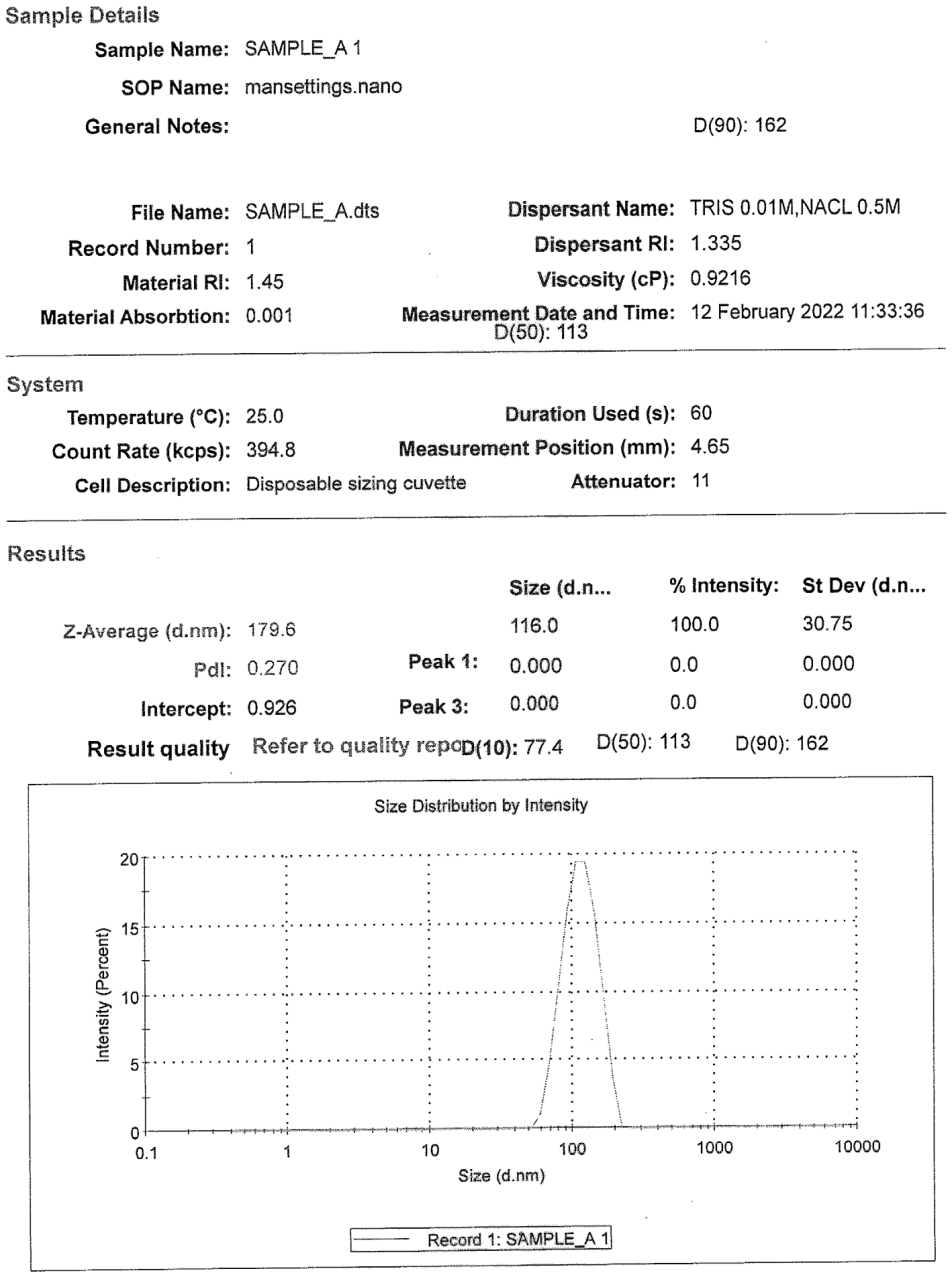
A representative size distribution graph with particle size populations using dynamic light scattering, D (50) = 113 nm and D (90) = 162 nm.

The DLS analysis for all the drug substance batches was performed. As per the analysis, the particle size distribution of all the DS batches was found to be in the range of 100–200 nm [D (90) analyses]. Each of the samples showed the presence of a single peak with a narrow size distribution range, indicating the absence of any aggregation of the virus particles (**Figure 2**).

### CoviWall immunization generates a potent humoral response in mice

**Figure 3:**
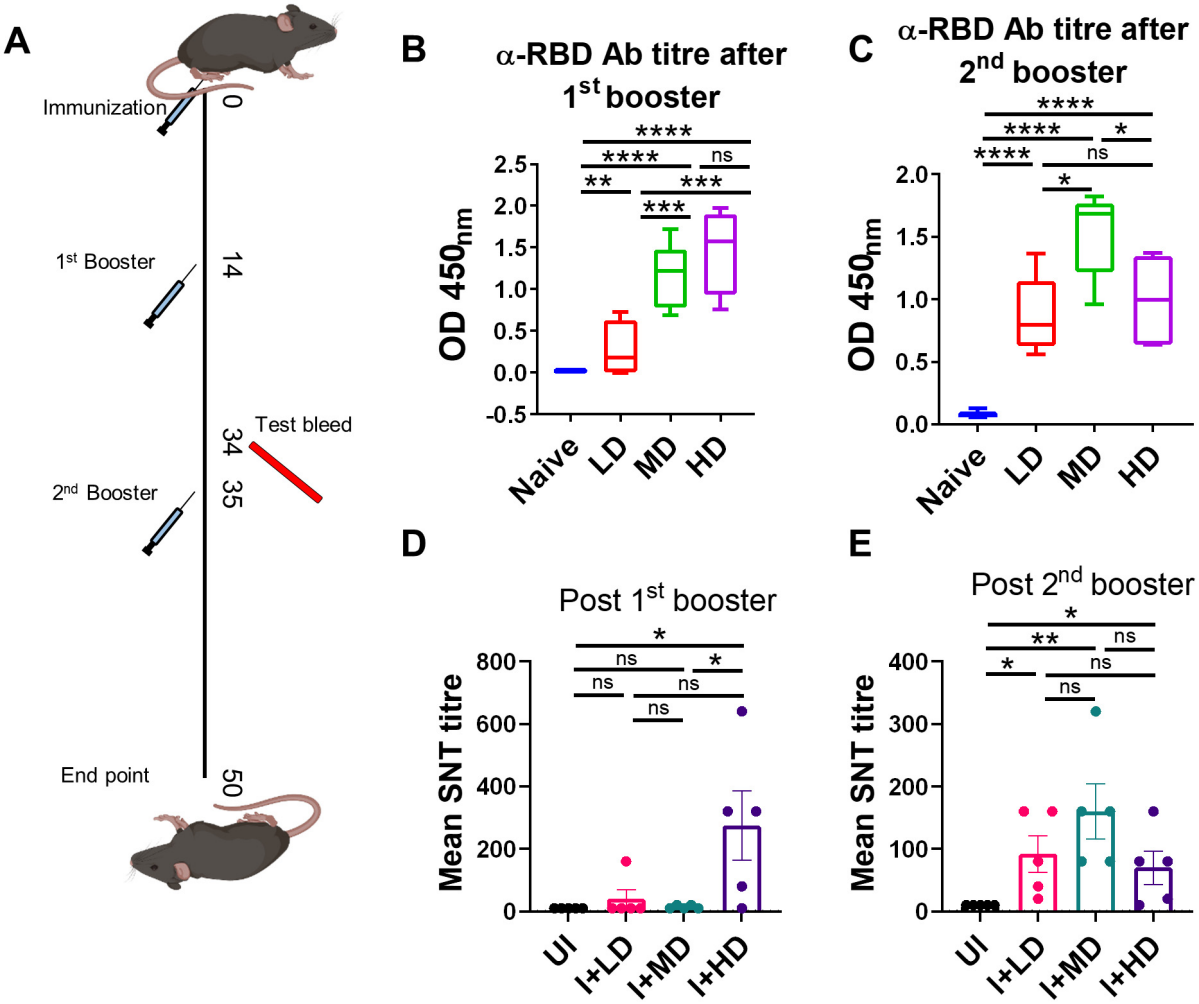
Evaluation of humoral response of CoviWall-immunized C57BL/6 mice. C57BL/6 mice were intramuscularly immunized with CoviWall [low dose (LD), mid dose (MD), and high dose (HD)] followed by two booster doses on day 14 and day 35. The immunized and naïve animals were test bled through retro-orbital vein on day 34 (after the first booster) and day 50 (after the second booster) to evaluate the humoral response against SARS-CoV-2. **(A)** The schematic representation of the experimental design, immunization schedule, and test bleed time point. Ancestral RBD (α-RBD) antibody response evaluated by ELISA from the serum samples after **(B)** the first booster dose **(C)** the second booster dose. Neutralizing antibodies response was evaluated by SNT method from the serum samples after **(D)** the first booster dose and **(E)** the second booster dose. Bar graphs are plotted as mean ± SEM. For each experiment N = 5. One-way ANOVA using non-parametric Kruskal–Wallis test for multiple comparisons. *P < 0.05, **P < 0.01, ***P < 0.001, and ****P < 0.0001. ns, non-significant.

Humoral response against SARS-CoV-2 has been previously shown to be a good indicator of vaccine efficacy (19–21). We evaluated the humoral response in terms of anti-RBD antibody generation and neutralization of live virus *in vitro* in C57BL/6 mice immunized with low dose (LD), MD, and HD of CoviWall followed by two booster doses at day 14th and 35th. Test bleed was performed on day 34th and day 50th (after first immunization) and the humoral response was evaluated in the serum samples of immunized vs. unimmunized (naïve) mice (**Figure 3A**). We used ancestral RBD because Wuhan-RBD is regarded as a reference for other variants with acquired mutations. Our ELISA data suggest a significant titer of anti-RBD antibodies in both MD- and HD-immunized mice even after the first booster dose, which was maintained/enhanced after the second booster dose. Notably, the LD-immunized mice also showed a significant rise in anti-RBD antibody titer after the second booster dose, suggesting that a single booster dose was insufficient and a second booster dose was needed to achieve significant anti-RBD antibody titer in LD-immunized mice (**Figures 3B, C**). Interestingly, a single booster dose failed to generate the serum-neutralizing antibody (SNT) response in LD- and MD-immunized mice and only showed a significant SNT titer (>1:100–200) after the second booster dose (**Figures 3D, E**). Taken together, our data show robust humoral response in CoviWall-immunized mice, which was found to be optimal after the second booster dose.

### CoviWall immunization induces Th1 cell–skewed adaptive response in mice

**Figure 4:**
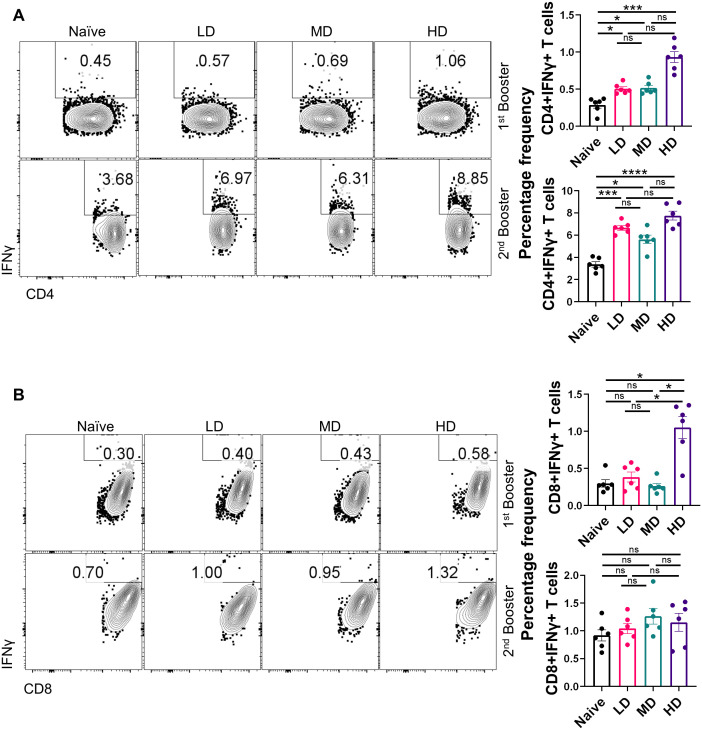
Evaluation of T-cell response of CoviWall-immunized C57BL/6 mice. T-cell response was studied in intramuscularly CoviWall-immunized [low dose (LD), mid dose (MD), and high dose (HD)] mice after the first (top panel) and second booster (bottom panel) doses by evaluating IFN-γ response in **(A)** CD4+ T and **(B)** CD8+ T cells from splenocytes. The figure shows representative FACS contour plot (left) and bar graph plotted as mean ± SEM. For each experiment N = 5. One-way ANOVA using non-parametric Kruskal–Wallis test for multiple comparisons. *P < 0.05, ***P < 0.001, and ****P < 0.0001. ns, non-significant.

Several lines of evidence now suggest that not only the humoral response is important in the context of vaccine immunity but also the cellular immune response defined by T helper cells (6, 9, 22). Indeed, studies have shown that vaccines continue to protect T-cell immunity even if there is a diminished antibody response against SARS-CoV-2 variants. Moreover, Th1-induced IFN-γ response is regarded as a reliable correlate of protection (23–25). To understand the T-cell response in the CoviWall-immunized mice, we used splenocytes from the first booster dose and the second booster dose and stimulated with ancestral RBD *in vitro* for 72 h and then performed intracellular cytokine staining for evaluating the effector T-cell response. The gating strategy used for the analyzing the frequency of IFN-γ+, IL-4+, and IL-17A+ cells in the CD4 and CD8 compartment is shown in **Supplementary Figure S1**. Our data indicates that the first booster dose was only sufficient to induce Th1 & Tc1 response in HD-immunized mice but not in the MD and LD groups. The HD-immunized mice, but not MD and LD, showed a 3.5- to 4-fold increase in the frequency of IFN-γ+CD4+ T cells as well as IFN-γ+CD8+ T cells after the first booster dose as compared to naïve group (**Figures 4A, B**, top panel). Notably, the Th1 response was enhanced in LD- and MD-immunized mice after the second booster dose, suggesting that LD and MD immunization requires a second booster dose for optimal Th1 response, however, the HD group showed saturated Th1 response achieved even after the first booster dose (**Figure 4A**, bottom panel). Moreover, the IFN-γ+CD8+ T-cell response was not further boosted after the second booster dose (**Figure 4B**, bottom panel). We also evaluated the Th2 and Th17 response in the immunized mice. Both Th2 and Th17 cell responses are not good correlates of protection but are important in the context of COVID-19 severity. Our data show no significant changes in the Th2 and Th17 cell response in immunized mice as compared to those in naïve mice (**Supplementary Figures S2**, **S3**). Taken together, CoviWall-immunized mice induces strong Th1 cells response suggesting protection against Delta strain infection.

### CoviWall immunization protects Syrian hamsters against B.1.617.2 strain infection

**Figure 5:**
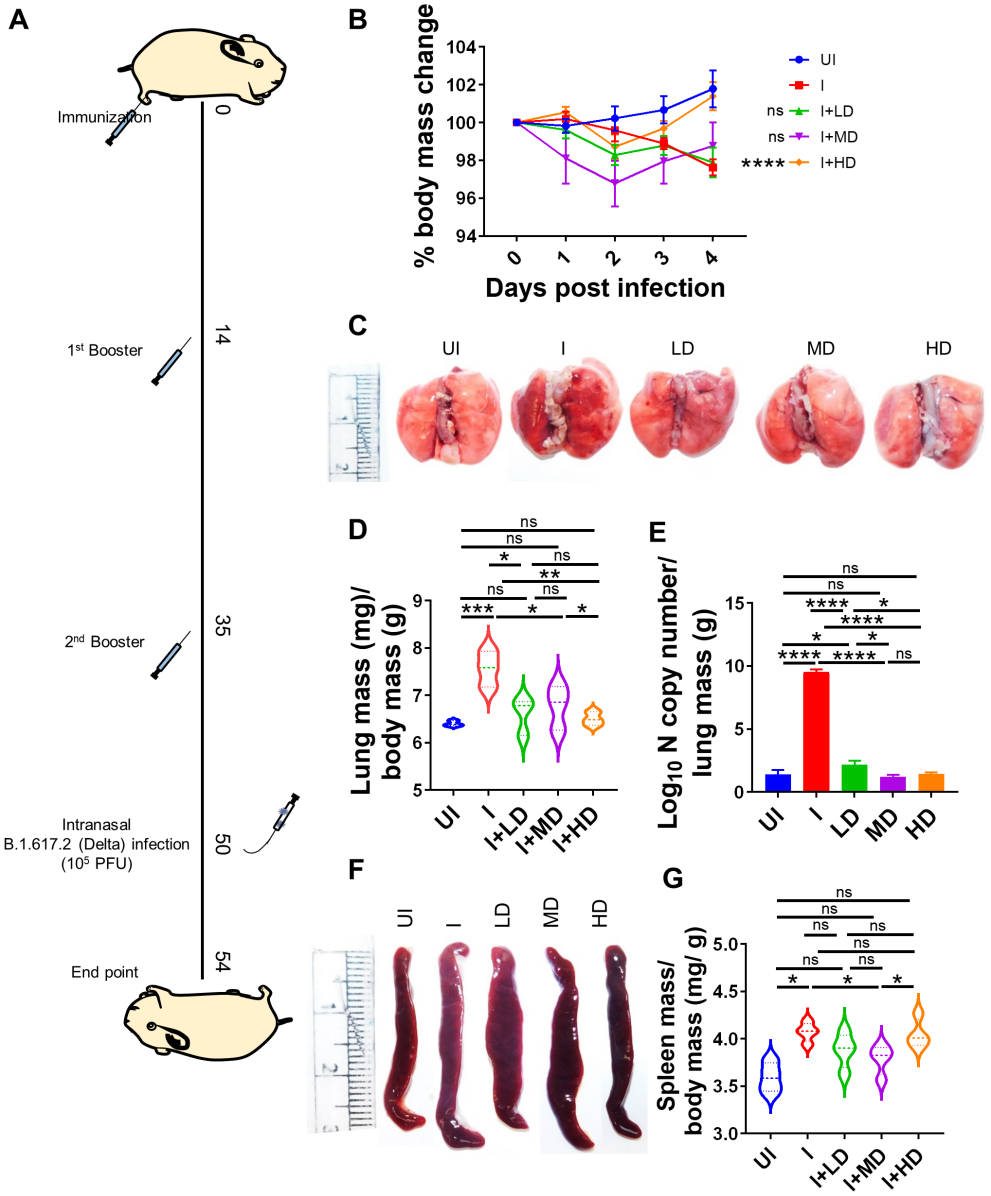
Protective efficacy of CoviWall in Syrian hamster. Protective efficacy of CoviWall was evaluated in Syrian hamster model of SARS-CoV-2 infection. **(A)** Schematic representation of the experimental design. Briefly, the hamsters were divided into five groups, viz., uninfected (UI), infected (I), and three intramuscularly immunized groups that received three immunization doses of CoviWall as low dose (I + LD), mid dose (I + MD), and high dose (I + HD). All the animals except UI was given intranasal challenge with B.1.617.2 strain on the 50th day after immunization. All the animals were monitored for changes in body mass and activity after challenge and were euthanized on 4 days postinfection (dpi). **(B)** Percentage change in body mass as compared to day 0 body mass of that group was plotted as line graph as mean ± SEM. **(C)** Representative image of excised lungs of Syrian hamsters at 4 dpi. **(D)** Lung mass/body mass ratio. **(E)** Lung viral load at 4 dpi. **(F)** Representative image of spleen. **(G)** Spleen mass/body mass ratio. For each experiment N = 5. One-way ANOVA using non-parametric Kruskal–Wallis test for multiple comparisons. *P < 0.05, **P < 0.01, ***P < 0.001, and ****P < 0.0001. ns, non-significant.

Since we observed robust SARS-CoV-2–specific humoral and T-cell response in CoviWall-immunized mice, we reasoned that CoviWall immunization would also protect against COVID-19 pulmonary pathology. To understand the protective efficacy of CoviWall immunization, we used the Syrian hamster model for SARS-CoV-2 infection. Syrian hamster model along with hACE2 transgenic mice model are the two most routinely used animal models for pre-clinical trials (26–30). Previous studies including studies from our group have shown that intranasal SARS-CoV-2 infection in Syrian hamsters leads to high pulmonary viral load and histopathology at day 4 after challenge, which could be used as the optimized time point to study the protective efficacy of vaccinated hamsters (31–33). Syrian hamsters were immunized with LD, MD, and HD and given two booster doses, similar to the mice immunization strategy; thereafter, all the animals, except the uninfected (UI) group, were given an intranasal challenge with B.1.617.2 strain under mild anesthesia on the 50th day. All the animals were monitored until day 4 after infection and sacrificed on day 54 to evaluate the clinical parameters associated with COVID-19 (**Figure 5A**). In terms of body mass loss, hamsters immunized with MD and HD (i.e., I + MD and I + HD, respectively) showed a body mass gain trend after 2 days post-infection (dpi) with the I + HD group, showing 3%–4% body mass gain as compared to the I control (**Figure 5B**). Previous studies have shown lung inflammation and splenomegaly as an indicator of infection severity in hamsters. In line with this, we found significantly reduced lung inflammation and lung mass/body mass ratio in the immunized hamsters in comparison to the I unimmunized animals (**Figures 5C, D**). Notably, we found highly reduced viral load, reduced to baseline, indicating efficient virus clearance in the lungs of immunized animals (**Figure 5E**). Furthermore, splenomegaly condition was also found to be mitigated in the I + LD and I + MD groups in comparison to the I group (**Figures 5F, G**). Together, clinical manifestations, pulmonary inflammation and viral load were significantly reduced in immunized hamsters, suggesting strong protective efficacy in hamsters.

### Mitigation of pulmonary pathology in CoviWall-immunized Syrian hamster

**Figure 6:**
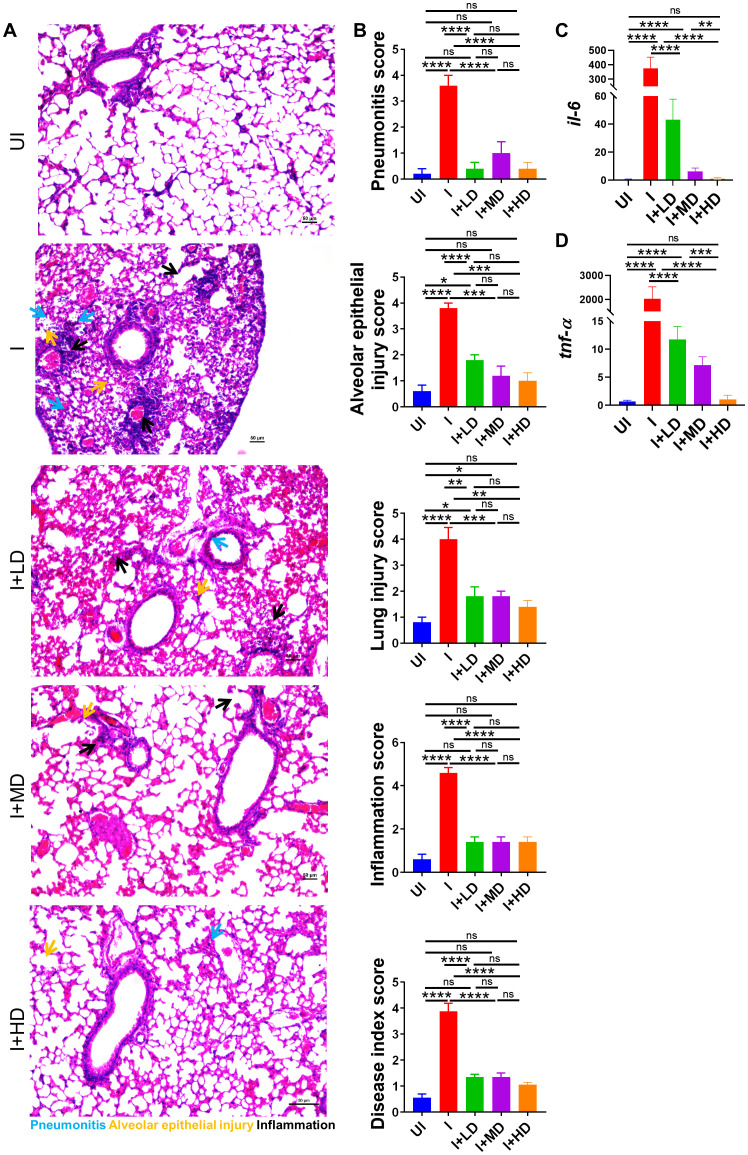
Histopathological evaluation of CoviWall-immunized Syrian hamster against B.1.617.2 strain. Left lower lobe of the lungs was fixed in 10% formalin and paraffin embedded, sectioned, and stained with HE stain. The HE-stained sections were imaged at ×10 and histological assessment was performed in a blinded manner by trained pathologist on the scale of 0–5 as described in the methodology section. **(A)** Representative HE-stained lung image showing regions of pneumonitis (blue arrow), alveolar epithelial injury (yellow arrow), and inflammation (black arrow). **(B)** Bar graph showing assessment score for pathological features as mean ± SEM. mRNA expression of pro-inflammatory cytokines genes were done from the lung samples. **(C, D)** Bar graph showing relative mRNA expression of IL6 and TNF-alpha as mean ± SEM respectively. For each experiment N = 5. One-way ANOVA using non-parametric Kruskal–Wallis test for multiple comparisons. *P < 0.05, **P < 0.01, ***P < 0.001, and ****P < 0.0001. ns, non-significant.

SARS-CoV-2 infection is primarily an infection of the lungs and is known to cause local inflammation in the pulmonary region. COVID-19 leads to cytokine release syndrome, which results in acute respiratory distress syndrome in severe cases (34, 35). The induced local inflammation in the lungs leads to tissue injury and pneumonitis, which is an indicator of disease severity. To understand the pathophysiology of lungs of immunized vs. unimmunized animals, we carried out a detailed histopathological assessment of lungs by blinded random scoring by a trained pathologist. The Hematoxylin & Eosin (HE)-stained lung sections acquired at ×10 magnification show rich regions of pneumonia (blue arrow), alveolar epithelial injury (yellow arrow), and inflammation (black arrow) along with lung injury. The HE assessment score indicates one- to two-fold mitigation in lung injury score, alveolar epithelial cell injury score, and inflammation score in immunized animals as compared to the unimmunized animals (**Figures 6A, B**). Since, IL-6 and Tumor necrosis factor-alpha (TNF-α) levels are the major contributors of cytokine storm syndrome in the lungs during COVID-19, we also evaluated the mRNA expression of IL-6 and Tumor necrosis factor-alpha (TNF-α) in the vaccinated vs. unvaccinated hamsters. The mRNA expression of both IL-6 and TNF-α was found to be suppressed in the lungs of vaccinated hamsters as compared to unvaccinated infection control. Furthermore, the highest suppression in the mRNA expression was seen in the HD-vaccinated hamsters as compared to that in the LD vaccinated hamsters, indicating that HD is the optimal and sufficient dose to mitigate the levels of these pro-inflammatory cytokines (**Figures 6C, D**).

### High SARS-CoV-2 neutralizing antibody titer in CoviWall-immunized Syrian hamster

**Figure 7:**
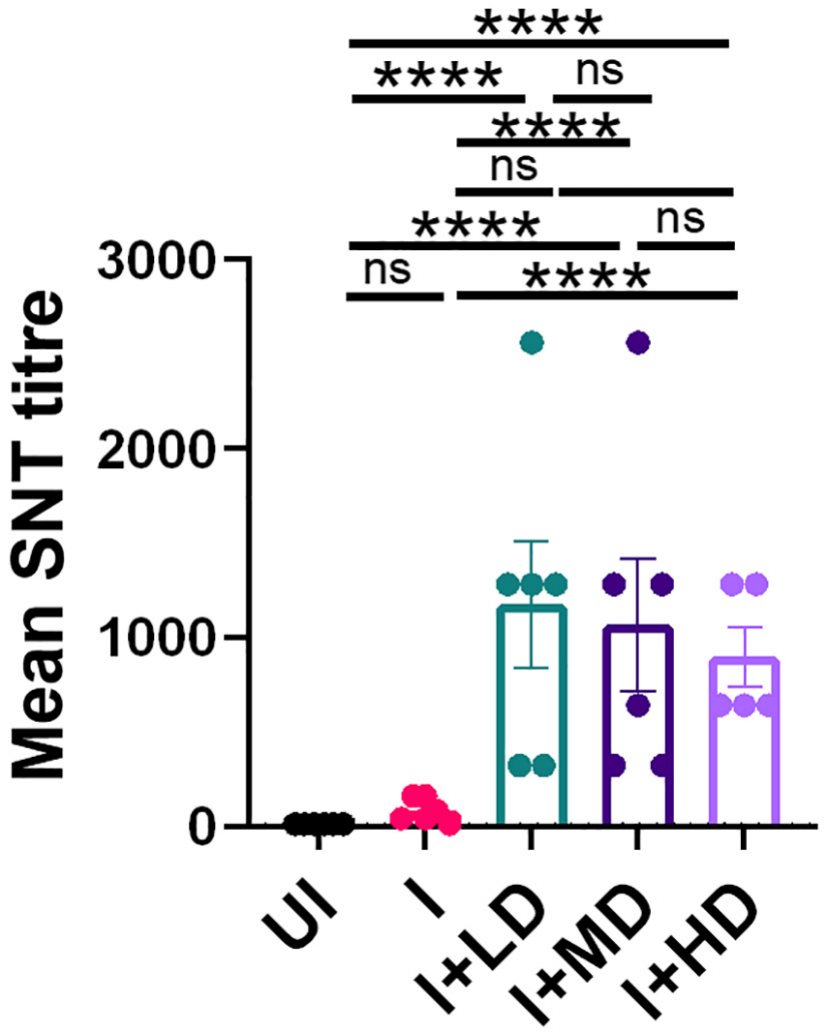
Neutralizing antibody response of CoviWall-immunized Syrian hamster. Serum samples at end point from the challenge study were used to evaluate the neutralizing antibody titer to understand the antibody correlates of protection. Bar graph showing mean ± SEM values of SARS-CoV-2 neutralizing antibody titer in the end point serum samples. For each experiment N = 5. One-way ANOVA using non-parametric Kruskal–Wallis test for multiple comparisons. ****P < 0.0001.

To finally evaluate the immune correlates of protection in challenged hamsters, we studied the SNT response generated in immunized vs. unimmunized hamsters in the context of B.1.617.2 infection. The SNT results show a significantly high neutralizing antibody titer in the serum samples of the I + LD, I + MD, and I + HD groups, which was similar in trend with the SNT titer obtained in immunized mice (**Figure 7**). Taken together, we also observed significantly elevated neutralizing antibody response in challenged hamsters, which corroborates well with the mice immunogenicity data.

## Discussion

The global rise of COVID-19 initiated efforts for vaccine and antiviral drugs that could be used to control and limit disease related morbidity and mortality. Delta (B.1.617.2) strain that was responsible for second wave of infection affected India and many other countries, severely owing to its high rate of transmission when compared to the Alpha (B.1.1.7), Beta (B.1.351), and Gamma (P.1) variants (4, 7, 8). Here, we report the development, immunogenicity, and protective efficacy of CoviWall, a whole-virion–inactivated B.1.617.2 (Delta strain), in mice and hamster model. CoviWall was designed, conceptualized, and developed by Panacea Biotec Ltd. in collaboration with Translational Health Science and Technology Institute (THSTI) to fight Delta wave surge in India and any such future needs as a measure of our preparedness. Three doses of CoviWall were used to immunize mice, namely, LD, MD, and HD. The complete immunization regime involves initial immunization dose followed by two booster doses at day 14 and day 35, respectively. The immunized mice showed high anti-RBD and neutralizing antibody titer and showed a Th1-skewed cellular response. Moreover, immunized hamsters that received intranasal B.1.617.2 challenge developed attenuated clinical and pulmonary pathology with diminished lung viral load and lower mRNA expression of IL-6 and TNF-α, suggesting an overall robust protective immunity by CoviWall.

SARS-CoV-2 infection, which was first reported in Wuhan, China, in December 2019 acquired mutations in its genome, which led to the rise of new variant strains (21). Of particular interest are the mutations in the RBD and the N-terminal domain (NTD) such as the N501Y mutation of RBD, which is found in all variants except the Delta variant and has increased the spike protein affinity to ACE2 receptor by increasing viral binding and entry (36); whereas the Delta (B.1.617.2) variant, in addition to the D614G spike mutation, contains the following mutations in RBD (L452R and T478K), NTD (R158G, T19R, G142D, Δ156-157), S2 region (D950N), and a mutation at the site close to furin cleavage site. These mutations not only increased the replication efficiency and reduced the neutralizing antibody response but also increased the transmissibility of the Delta variant (4, 8). Previous efforts in vaccine effectiveness against SARS-CoV-2 infection using inactivated vaccines like BBV152 (Covaxin) and other vaccines during a delta-driven surge were found to be not that successful (9). The high transmissibility, infectivity, and virulence of the delta variant, which causes severe disease, might have contributed to a reduced vaccine effectiveness against symptomatic infections, which has been reported to be as low as 56% for inactivated and other vaccines in multiple studies worldwide (9). It was, therefore, a pressing need to formulate an effective vaccine that would protect against B.1.617.2 (Delta) variant and any such future needs as a measure of our preparedness. With this rational, we manufactured a whole-virion–inactivated vaccine based on B.1.617.2 variant, which was developed in a manner similar to Covaxin. The idea was to generate a vaccine using a process that is robust, consistent, and quickly adaptable for production under cGMP (15–17, 37). Three consistency batches were generated under cGMP, which matched the set specifications. Given the fact that Covaxin was based on ancestral Wuhan strain inactivation, we reasoned that CoviWall, based on the B.1.617.2 strain inactivation, could have great potential in providing protection against B.1.617.2 strain infection—the dominant and most fatal strain at the time.

To understand the immunogenicity and protective efficacy of CoviWall, we carried out pre-clinical animal studies in mice and Syrian hamsters, which broaden the perspective on vaccine efficacy. Mice are commonly used in vaccine efficacy studies to conduct dose-response evaluations, assessing various dosing regimens and their effects on the immune response (38, 39). This is crucial for optimizing vaccine formulations before human trials (40). Here, we used mice to characterize humoral and cellular immune responses. The data gathered are critical for regulatory submissions, as agencies require evidence of the safety and efficacy of the CoviWall vaccine for phase-I clinical trials. For assessing the immune correlates of protection, we evaluated the anti-RBD antibody titer and neutralizing antibody titer from the serum samples of immunized mice and compared them with naïve mice, sera negative for antigen. It is worth noting that we used native/ancestral/wild-type RBD protein to evaluate the anti-RBD antibody titer as well as cellular T-cell response (later on). The rationale for using wild-type RBD for evaluating the humoral and cellular T-cell response stems from the fact that wild-type RBD shares greater homology with other VoCs and could be used as a baseline to extrapolate the protective efficacy in emerging variants. The mice immunized with CoviWall showed significantly high levels of anti-RBD and neutralizing antibody response both of which have been previously reported as recommended correlates of protection against currently predominant infection strain as well as future emerging variants. Similarly, we also evaluated the T-cell response in immunized mice by intracellular cytokine staining flow cytometry. IFN-γ cytokine, which is majorly contributed by Th1 cells and, to a lesser extent, by CD8+ T cells, is crucial for viral infection. Several lines of evidence have previously shown that a Th1-biased immune response is crucial in the context of effective immunity against viral infections. Indeed, RBD-specific IFN-γ response has been previously shown to be a reliable indicator of protection against SARS-CoV-2 infection and has been used as a gold standard parameter for evaluating vaccine efficacy (23, 36, 41). It is important to point out here that, although we did found robust humoral and Th1 response against wild-type RBD, we did not use spike protein or other variants RBD protein due to the limitation of resources, and the immunogenicity of CoviWall against spike or other variants RBD needs to be evaluated for proof of concept for protection against emerging VoCs.

To study the protective efficacy of CoviWall vaccine, we used Syrian hamster model for challenge study. Syrian hamster model is a reliable, reproducible, and harmonized animal model for SARS-CoV-2 intranasal challenge and has been routinely used for COVID-19 pre-clinical vaccine trial studies. Hamsters have SARS-CoV-2 infection usually causes mild to moderate disease, allowing to evaluate vaccine effectiveness in preventing symptomatic illness and severe outcomes. The immune response in hamsters, including neutralizing antibodies and T-cell responses, closely resembles that in humans, aiding in the extrapolation of findings to human COVID-19 vaccine responses (29, 31, 42–44). Hamsters receiving complete dose of CoviWall immunization were largely protected against B.1.617.2 intranasal challenge with diminished lung viral load and pulmonary pathology. It is noteworthy that B.1.617.2 infection has been reported to cause 1,000 times higher lung viral load and is regarded as highly pathogenic VoC. Remarkably, CoviWall-immunized hamsters not only showed withdrawal of COVID-19 manifestations but also showed efficient clearance of viral load from the lungs in all the three doses of CoviWall (LD, MD, and HD), suggesting a strong overall protective efficacy of CoviWall. Given aggressive infectivity of B.1.617.2 strain, we reason that CoviWall immunization may have good protective efficacy against emerging variants such as Omicron and Omicron sub-lineages; however, to get a holistic picture, more experiments aimed at determining the immunogenicity and protective efficacy of CoviWall against VoCs need to be carefully designed and evaluated. In summary, we report here pre-clinical animal study data of CoviWall, a whole-virion–inactivated B.1.617.2, showing robust humoral and Th1 response forming the basis of strong protective efficacy. This study, therefore, demonstrates the development of a consistent process for the manufacture of a cGMP grade SARS-CoV-2 vaccine against a deadly strain such a B.1.617.2 and its effectiveness in a SARS-CoV-2–specific animal model, auguring well for its further development in clinical trials.

## Materials and methods

### Description of the manufacturing process

Whole-virion–inactivated coronavirus (SARS-CoV-2) vaccine drug substance is a mammalian cell culture–based vaccine. The continuous cell line used for manufacturing is Vero-based. The vaccine virus strain of SARS-CoV-2 was Delta variant (Lot no. 70045238) of characterized live coronavirus (SARS-CoV-2) (Isolate hCoV-19/USA/PHC658/2021 (Lineage B.1.617.2; Delta Variant) NR-55611 from St. Jude Children’s Research Hospital by Dr. Richard Webby and Dr. Anami Patel.

The MVS of strain of live coronavirus (SARS-CoV-2) (Isolate hCoV-19/USA/PHC658/2021 (Lineage B.1.617.2; Delta Variant) was prepared at vaccine drug substance facility of Panacea Biotec Ltd. (PBL), Delhi. The sequence identity of the strain was confirmed by Next-Generation Sequencing (NGS) of Complete Genome Using Illumina^®^ iSeq™ 100 platform, whereas its titer was evaluated by TCID_50_ assay in Calu-3 cells by observation for CPE (5 days at 37°C and 5% CO_2_).

The manufacturing process of whole-virion–inactivated coronavirus (SARS-CoV-2) vaccine drug substance comprised of many major steps, including the revival of Vero cells followed by a virus infection, harvest, purification, and ends with filtration of the drug substances. At first, cells were cultivated to generate enough substrate cells (4,000 to 8,000 million cells) during the cell propagation phase. The cell growth medium used was Dulbecco’s Modified Eagle Medium (DMEM) or any other media that supports Vero cell growth supplemented with Fetal bovine serum (FBS) and glutamate. After revival, the propagation of the cells was performed in five 10-layered tissue culture grade flasks/cell factories (CF-10). Flasks were incubated in a 37°C humidified incubator with 5% to 8% CO_2_.

The cells were then infected to initiate the virus replication phase (3–4 days) in a biosafety level 3 (BSL-3) facility. The quantum of virus inoculum required was calculated on the basis of the TCID_50_ value (titer) of the virus stock, Multiplicity of infection (MOI), and total number of cells. The MOI was maintained at 0.01 to 0.5. Once the virus production was completed until the point of harvest and the CPE was observed, the downstream process was started by harvesting the culture. At this stage, a sample was also collected and tested for virus titer (TCID_50_).

The virus was then inactivated by BPL for 24–30 h. After BPL neutralization, a sample was collected and tested for virus inactivation. Inactivation kinetics was performed as per protocol no. ARD/D/CoV/001 in two different aliquots. The study employed two methods for inactivation with multiple sampling time points (16, 20, 24, and 36 h). All the time-dependent samples were tested for residual live virus test as per protocol no. ARD/D/CoV/002. Endonuclease treatment of the inactivated virus harvest was performed in order to digest the host cell DNA into smaller fragments that could be eliminated in further processing steps. This was followed by steps of clarification to remove cell debris, affinity chromatography, and concentration and further diafiltered to eliminate low–molecular weight impurities as well as to reduce the volume for next step. Finally, a filtration step was performed to reduce the bioburden.

The flowchart depicted below summarizes the manufacturing process for whole-virion–inactivated coronavirus (SARS-CoV-2) vaccine drug substance: 


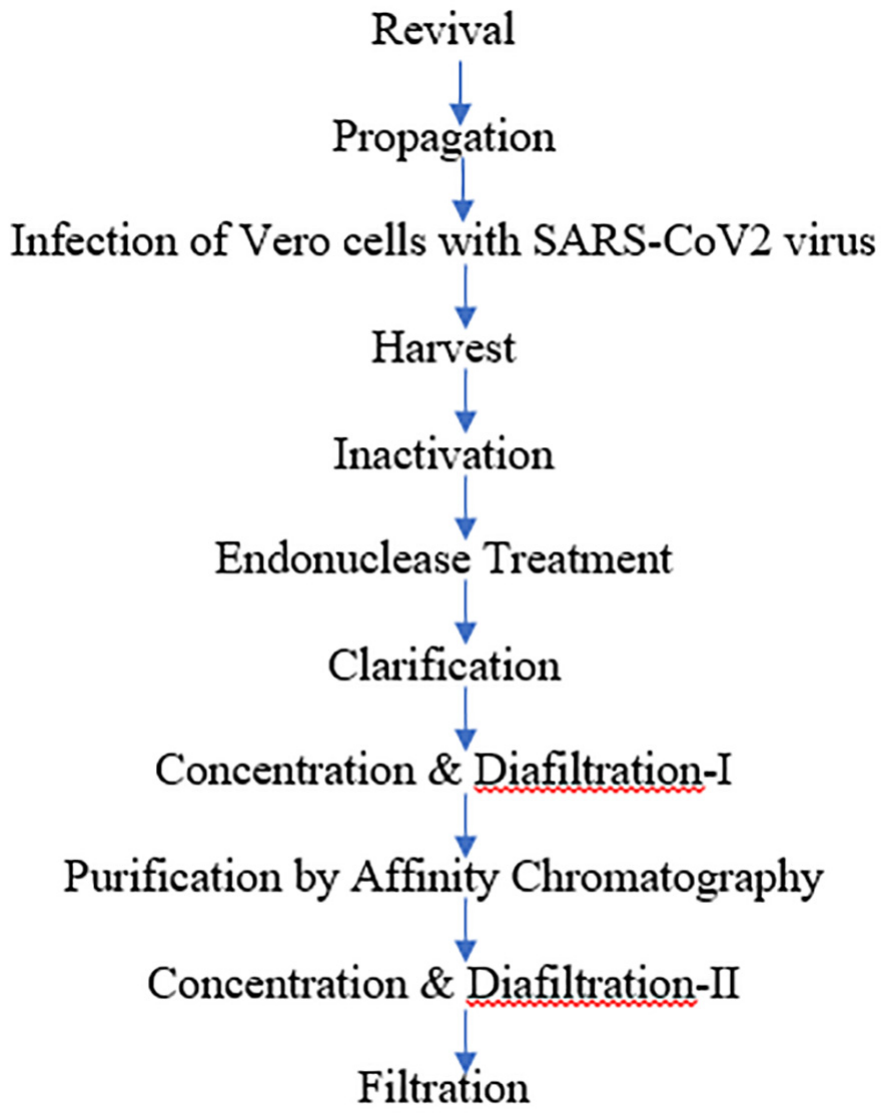


Three individual consistency batches (DLT 2104, DLT 2105, and DLT 2106) were generated under GMP conditions using the above process.

### Analytical testing for whole-virion–inactivated SARS-CoV-2 virus bulk/drug substance

Analytical testing was performed as per the specifications of the whole-virion–inactivated SARS-CoV-2 virus bulk/drug substance to comply for its criteria for description, virus inactivation, identity, potency, purity, and other physical, chemical, and microbiological properties relevant to the clinical use of the product.

Identity test was performed using a SARS-CoV2 (2019-nCoV) Spike Detection ELISA kit (Sino Biologicals, Cat. No. KIT40591).

In-process samples were also tested for tests related to identity, purity, and potency (total protein) in order to analyze step-wise and stage-specific process yields and efficiency. Residual impurity Residual host cell proteins (HCPs), residual host cell DNA (HCD), trypsin content, endonuclease, BPL, and Bovine serum albumin (BSA) content were estimated.

Three consistency batches for the drug substance were taken under the cGMP guidelines with reproducible results, which indicated the robustness of the process.

### Formulation

The drug product, i.e., inactivated SARS CoV-2 vaccine (whole virion, adjuvanted) is a sterile uniform suspension (after shaking) free from any extraneous particles. The vaccine contained whole-virion–inactivated SARS CoV-2 antigen along with aluminium hydroxide gel and TLR 7/8 agonist (imidazoquinolinone) and further suspended in phosphate buffer saline solution to which 2-phenoxyethanol was added as a preservative. The vaccine was presented in United States Pharmacopeia (USP) type 1 glass vials closed with bromobutyl rubber stoppers and flip-off seal. The quality of vaccine was controlled as per pre-defined specifications, which control identity, potency, purity, dose delivery, and other physical, chemical and microbiological properties relevant to the clinical use of the product.

### Western Blotting

The biological identity as well as epitope integrity of the virus (SARS-CoV-2) after BPL inactivation were confirmed by Western blot (WB). BPL is known to chemically modify amino acids and, at specific concentrations, can alter the antigenic potential of viruses (Gupta et al., 2021). The detectability of structural proteins [mainly S (S1 and S2) and N] by WB is hence used by several vaccine manufacturers as a readout for optimal inactivation, ensuring antigenicity of the drug substance (Ganneru et al., 2021) (16).

### Dynamic Light Scattering

Particle size can be determined by measuring the random changes in the intensity of light scattered from a suspension or solution. This technique is commonly known as dynamic light scattering (DLS). When virus particles are emitted from the cells, the distribution parameters from a DLS experiment permit clear observation of the size distributions of virus particles in the presence of other substances. Comparative DLS analysis of the virus after inactivation is recommended to assess the virus aggregation of the drug substance.

### Animals and ethical approval

Forty C57BL/6 mice (6–8 weeks old and gender matched) bred and maintained at the Small Animal Facility (SAF) were used for the immunogenicity study, which was carried out in the procedure room of SAF. Thirty female Syrian hamsters (6–9 weeks old) were procured from the National Institute of Nutrition (NIN), quarantined at THSTI, and immunized at procedure room (SAF) and then shifted to BSL3 (THSTI) for conducting the challenge study. All the animals were maintained in a 12-h/12-h light/dark cycle and fed a standard pellet diet and water *ad libitum*. The study was carried out in strict accordance with the recommendations described in the NC3R guidelines and Institutional Animal Ethics Committee (IAEC) (THSTI) guidelines. All of the animal experiments were reviewed and approved by the Committee on the Ethics of Animal Experiments of THSTI (IAEC/THSTI/178), and animal challenge study was also approved by Institutional Biosafety Committee (IBSC) and The Review Committee on Genetic Manipulation (RCGM). All the animal challenge experiments were conducted in accordance with strict guidelines of IBSC.

### CoviWall immunization in C57BL/6 mice

Mice were divided into four groups, *viz*., naïve (mock immunization), LD (LD immunization; 1.2 μg), MD (MD immunization; 2.4 μg), and HD (HD immunization; 4.8 μg) groups, with each group containing five mice. The immunization dose was followed by two booster doses on day 14 and day 35, and immunogenicity was assessed in terms of antibody response (by ELISA and SNT) and T-cell response (by Fluorescence-activated cell sorting (FACS)) at day 34 and day 50 after first immunization, which was also the end point of the study. At the end point, the animals were euthanized, and their blood by cardiac puncture and spleen was excised and used further.

### B.1.617.2 challenge study in Syrian hamster

Six- to 9-week-old golden Syrian hamsters (mixed gender) were randomly grouped (n = 6 hamsters per group) as UI, infected (I), LD (1.2 μg) immunized with infection (I + LD), MD (2.4 μg) immunized with infection (I + MD), and HD (4.8 μg) immunized with infection (I + HD). The initial immunization dose was followed by two booster doses on day 14 and day 35. On the 49 after immunization, the animals were shifted to BSL3. B.1.617.2 variant SARS-CoV-2 challenge was performed by intranasal infection of live Isolate hCoV-19/USA/PHC658/2021 (Delta Variant) B.1.617.2 (NR-55611) 10^5^ PFU/100 μL/hamster or with DMEM in mock control with the help of catheter under mild injectable anesthetized [ketamine (150 mg/kg) and xylazine (10 mg/kg)] inside ABSL3 facility as previously described (29, 31, 32, 43). All the protocols related to the study were approved by IAEC (IAEC protocol no.: IAEC/THSTI/178), RCGM, and IBSC committee. The humane end point of the challenge study was set as greater than 25%–30% body weight loss as compared to the day 0 body mass.

### Clinical parameters for challenged hamsters

For mice experiment, the end point of the study was day 50 after first dose of CoviWall, while for hamster study the end point was 4 days after challenge, i.e., 54 days after first immunization dose. After challenge, the body mass of the hamsters was recorded, and percentage change in body mass was calculated according to the following formula:


$$\begin{array}{c} \text{Percentage body mass change }\\ =\;\left(\left(\left(\text{m}1\;-\text{ m}0/\text{m}0\right)\;\times \;100\right)\;+\;100\right) \end{array}$$

Here, 100 was added to normalize the values on the scale of 100. At the end point, which was day 4 after infection, all the animals were euthanized, and necropsy was performed to observe clinical changes in the gross morphology of lungs and spleen as well as their organ mass. Lungs were excised and imaged, and left lower lobe of lung was fixed in 10% neutral formalin solution and used for histopathological assessment by HE staining. The remaining lung was homogenized in 2 mL of TRIzol solution and used for RNA isolation for lung viral load estimation. The serum samples were collected from blood isolated by cardiac puncture and clotted at room temperature. These serum samples were head inactivated and used for serum neutralization test (SNT).

### Lung viral load

The isolated lung was homogenized in 2 mL of TRIzol reagent (Invitrogen), and RNA was isolated by TRIzol-Choloform method. Yield of RNA was quantitated by NanoDrop, and 1 µg of RNA was used to reverse-transcribed to cDNA using the iScript cDNA synthesis kit (Bio-Rad, #1708891) (Roche). Diluted cDNAs (1:5) were used for qPCR by using KAPA SYBR^®^ FAST qPCR Master Mix (5×) Universal Kit (KK4600) on Fast 7500 Dx real-time PCR system (Applied Biosystems), and the results were analyzed with SDS2.1 software (29, 32). Briefly, 200 ng of RNA was used as a template for reverse transcription–polymerase chain reaction (RT-PCR). The Centers for Disease Control and Prevention (CDC)-approved commercial kit was used for of SARS-CoV-2 N gene: 5′-GACCCCAAAATCAGCGAAAT-3′ (forward) and 5′-TCTGGTTACTGCCAGTTGAATCTG-3′ (reverse). Hypoxanthine-guanine phosphoribosyl transferase (HGPRT) gene was used as an endogenous control for normalization through quantitative RT-PCR. IL-6: 5′-GGACAATGACTATGTGTTGTTAGAA-3′ and 5′-AGGCAAATTTCCCAATTGTATCCAG-3′ and TNF-α: 5′-AGAATCCGGGCAGGTCTACT-3′ and 5′-TATCCCGGCAGCTTGTGTTT-3′ primers were used for mRNA relative expression profiling. The relative expression of each gene was expressed as fold change and was calculated by subtracting the cycling threshold (Ct) value of HGPRT (HGPRT-endogenous control gene) from the Ct value of the target gene (ΔCT). Fold change was then calculated according to the formula POWER(2,−ΔCT)*10,000 (45–47).

### Histological assessment

The lungs were fixed in 4% (v/v) paraformaldehyde solution for 72 h, and the paraffin sections (3–4 μm) were prepared routinely. Hematoxylin and eosin stains were used to identify histopathological changes in the lungs. Histological assessment was done by trained pathologists on the basis of a random-blinded scoring system where pathological features such as pneumonitis, inflammation, alveolar epithelial injury, and lung injury were randomly scored on the scale of 0–5 (where 0 score was given for lung section showing no or negligible pathology and 5 was given for highest/severe pathology). The histopathology of the lung tissue was observed by light microscopy. Images were captured using a LEICA Versa 200 and were processed using software HALO v3.1.1076.379.

### Flow cytometry and intracellular cytokine staining

Spleens were harvested from C57BL/6 at the end point as indicated above. The isolated spleen was then processed by disruption of the spleens through a 100-μm filter. Red blood cells were lysed by ACK lysis buffer for 20–30 s followed by washing. Cells were plated in a 96-well plate at 0.5 × 10^6^ cells/well in 200 μL of complete Iscove's Modified Dulbecco's Medium (IMDM) media and stimulated with RBD protein (5 μg/mL) for 72 h in a CO_2_ incubator at 37 °C. Five to six hours prior to harvesting, the Golgi stop (Sigma) was added to the cells for arresting protein transportation. After stimulation, surface markers were stained for anti-CD4 (BioLegend) and anti-CD8 (BioLegend) along with live dead stain for 15–20 min at room temperature in Phosphate buffered saline (PBS) with 1% FBS. Cells were then fixed in Cytofix and permeabilized with Perm/Wash Buffer using a BD Fixation Permeabilization solution kit and stained with anti-IL-17A (TC11-18H10.1, BioLegend), anti-IFN-γ (XMG1.2, BioLegend), and anti-IL-4 (11B11, BioLegend) antibodies diluted in Perm/Wash buffer. All antibodies were used in 1:500 dilutions. Flow cytometry was done using BD FACS Symphony, and data were analyzed with FlowJo software (48, 49).

### ELISA

ELISA plates (Corning) were coated overnight with SARS-CoV-2 RBD (2 μg/mL), recombinant protein in 0.05 M carbonate-bicarbonate buffer (pH 9.6), and blocked in 5% skim milk in PBS. Serum samples were two-fold serially diluted and added to each well. Plates were incubated with goat anti-mouse Immunoglobulin G-Horseradish Peroxidase (IgG-HRP) antibodies and developed with 3,3′,5,5′-Tetramethylbenzidine (TMB) substrate. Reactions were stopped with 2 M hydrochloric acid, and the absorbance was measured at 450 nm using a microplate reader (PerkinElmer, USA). The end point titers were defined as the highest reciprocal dilution of serum to yield an absorbance greater than two-fold of the background values. Antibody titer below the limit of detection was determined as half the limit of detection.

### Serum neutralization test

The serum of the animal to be tested was inactivated in a 56°C water bath for 30 min. Serum was successively diluted 1:8 to the required concentration by a two-fold series, and an equal volume of challenge virus solution containing 100 (Cell Culture Infectious Dose 50) CCID50 viruses was added. After neutralization in a 37°C incubator for 2 h, cell suspension (1.0 × 10^5^/mL) was added to the wells (0.1 mL/well) and cultured in a CO_2_ incubator at 37°C for 3–5 days. The Karber method (Ramakrishnan, 2016) by observing the CPE was used to calculate the neutralization end point (convert the serum dilution to logarithm), which means that the highest dilution of serum that can protect 50% of cells from infection by challenge with 100 CCID50 virus is the antibody potency of the serum. A neutralization antibody potency <1:20 is negative, whereas that of >1:20 is positive.

### Statistical analysis

GraphPad Prism 7.0 software was used for analyzing and plotting the results. Body mass, viral load, percentage frequency FACS, histological assessment, etc., were compared and analyzed by using one-way ANOVA with n = 5. P-value of less than 0.05 was considered statistically significant.

## Data Availability

The original contributions presented in the study are included in the article/**Supplementary Materials**. Further inquiries can be directed to the corresponding authors.

## Supplementary material

Supplementary Figure 1 Gating strategy for evaluating IFNy, IL4 and IL17+ T cell response from the spleen of immunized mice.

Supplementary Figure 2 Evaluation of Th2 cell response in CoviWall immunized C57BL/6 mice. Splenocytes from CoviWall immunized mice were used after 1^st^ booster (top panel) and 2^nd^ booster (lower panel) to evaluate Th2 response by evaluating CD4+IL4+ T cell frequency by FACS. (left panel) Representative FACS contour plot and (right panel) bar graph showing mean ± SEM percentage frequency. For each experiment N=5. One way-Anova using non-parametric Kruskal-Wallis test for multiple comparison. *P < 0.05.

Supplementary Figure 3 Evaluation of Th17 cell response in CoviWall immunized C57BL/6 mice. Splenocytes from CoviWall immunized mice were used after 1^st^ booster (top panel) and 2^nd^ booster (lower panel) to evaluate Th17 response by evaluating CD4+IL17A+ T cell frequency by FACS. (left panel) Representative FACS contour plot and (right panel) bar graph showing mean ± SEM percentage frequency. For each experiment N=5. One way-Anova using non-parametric Kruskal-Wallis test for multiple comparison. *P < 0.05.
