# Supplementary figures and images for: CoviWall, a whole-virion-inactivated B.1.617.2 vaccine candidate, induces potent humoral and Th1 cell response in mice and protects against B.1.617.2 strain challenge in Syrian hamsters
Source: Front Immunol. 2025 Jan 22;15:1447962. doi: 10.3389/fimmu.2024.1447962 (PMC11794485; doi:10.3389/fimmu.2024.1447962)

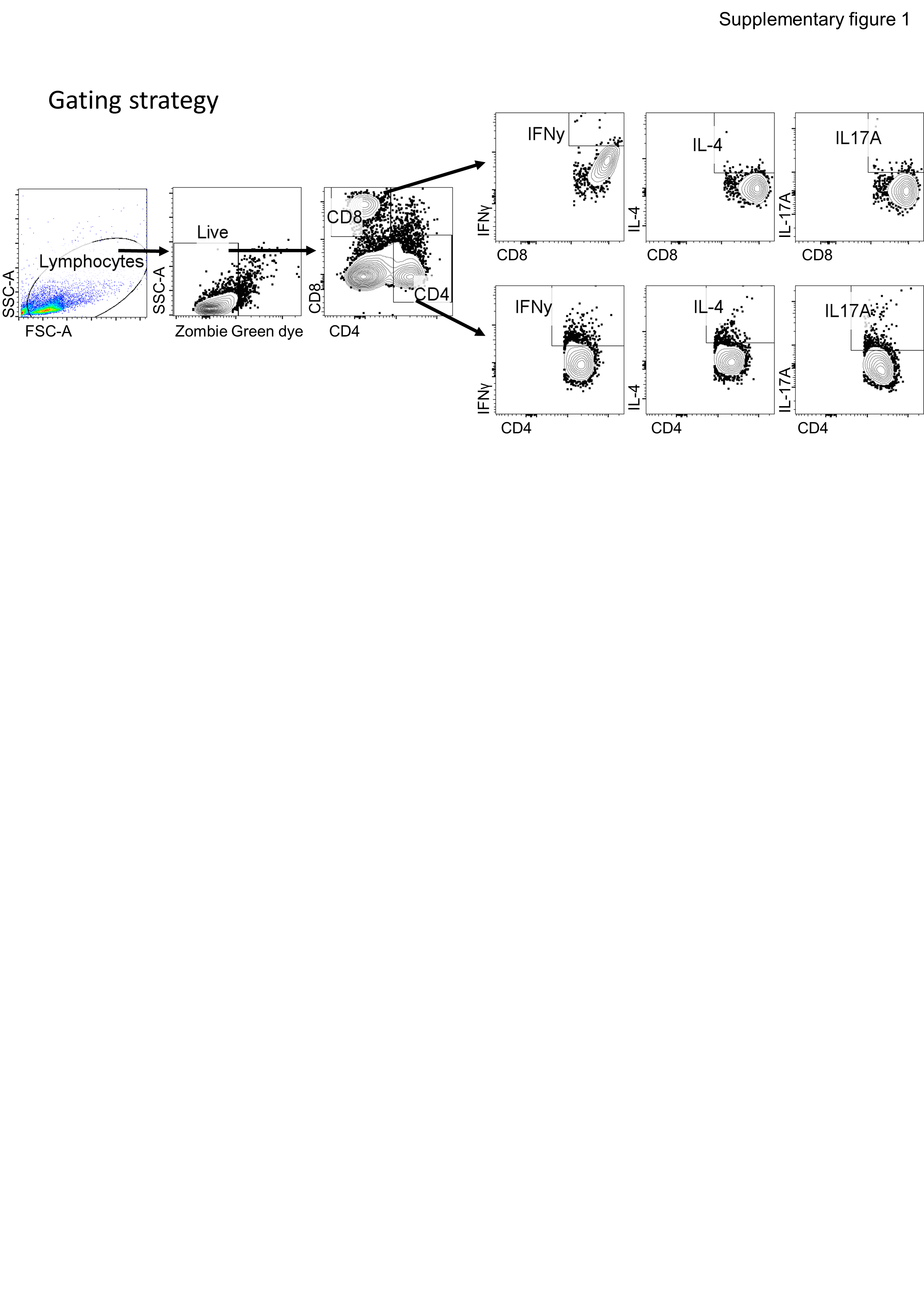

Supplement: Supplementary Figure 1 — Gating strategy for evaluating IFNy, IL4 and IL17+ T cell response from the spleen of immunized mice. [file Image1.tif]

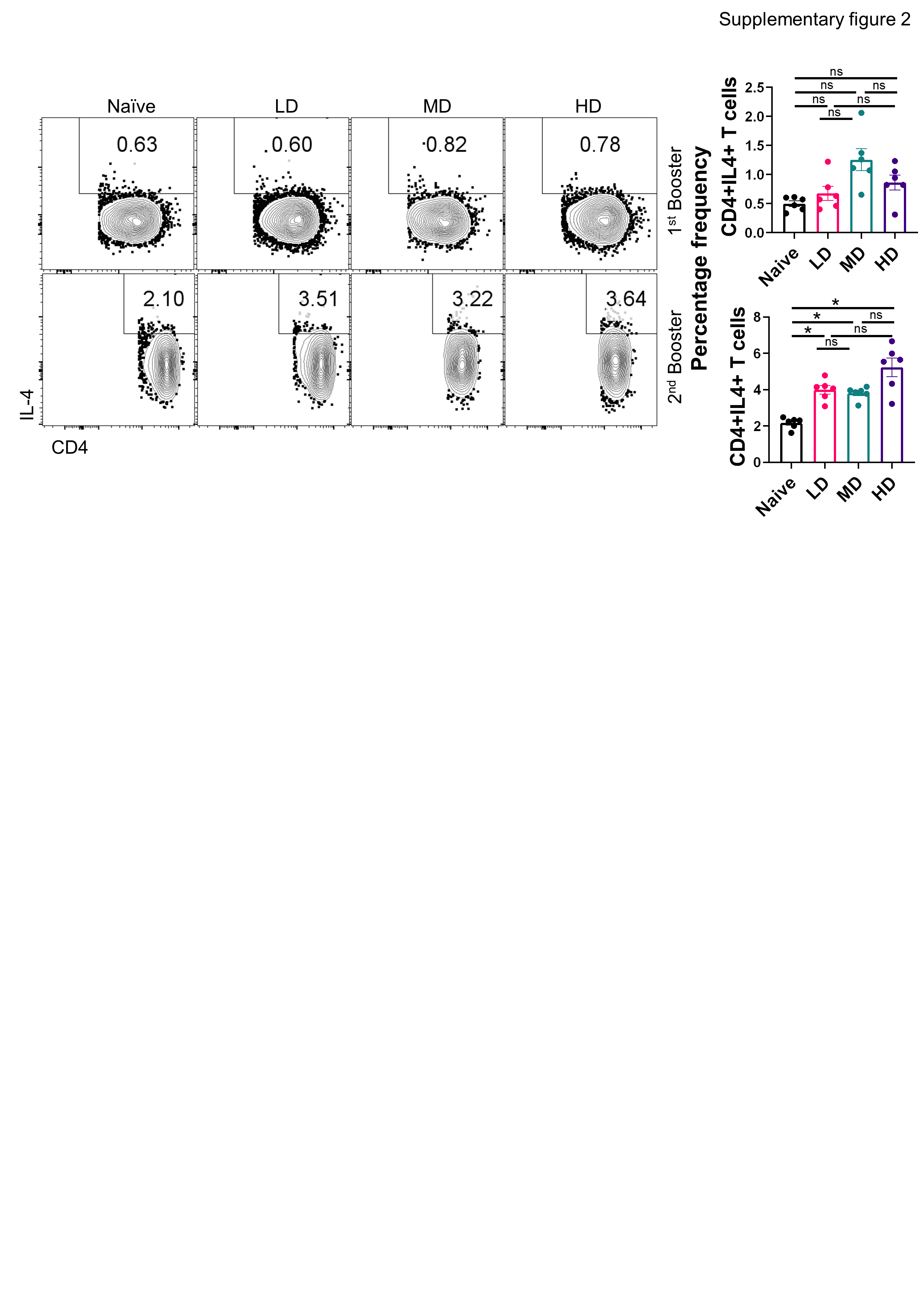

Supplement: Supplementary Figure 2 — Evaluation of Th2 cell response in CoviWall immunized C57BL/6 mice. Splenocytes from CoviWall immunized mice were used after 1st booster (top panel) and 2nd booster (lower panel) to evaluate Th2 response by evaluating CD4+IL4+ T cell frequency by FACS. (left panel) Representative FACS contour plot and (right panel) bar graph showing mean ± SEM percentage frequency. For each experiment N=5. One way-Anova using non-parametric Kruskal-Wallis test for multiple comparison. *P < 0.05. [file Image2.tif]

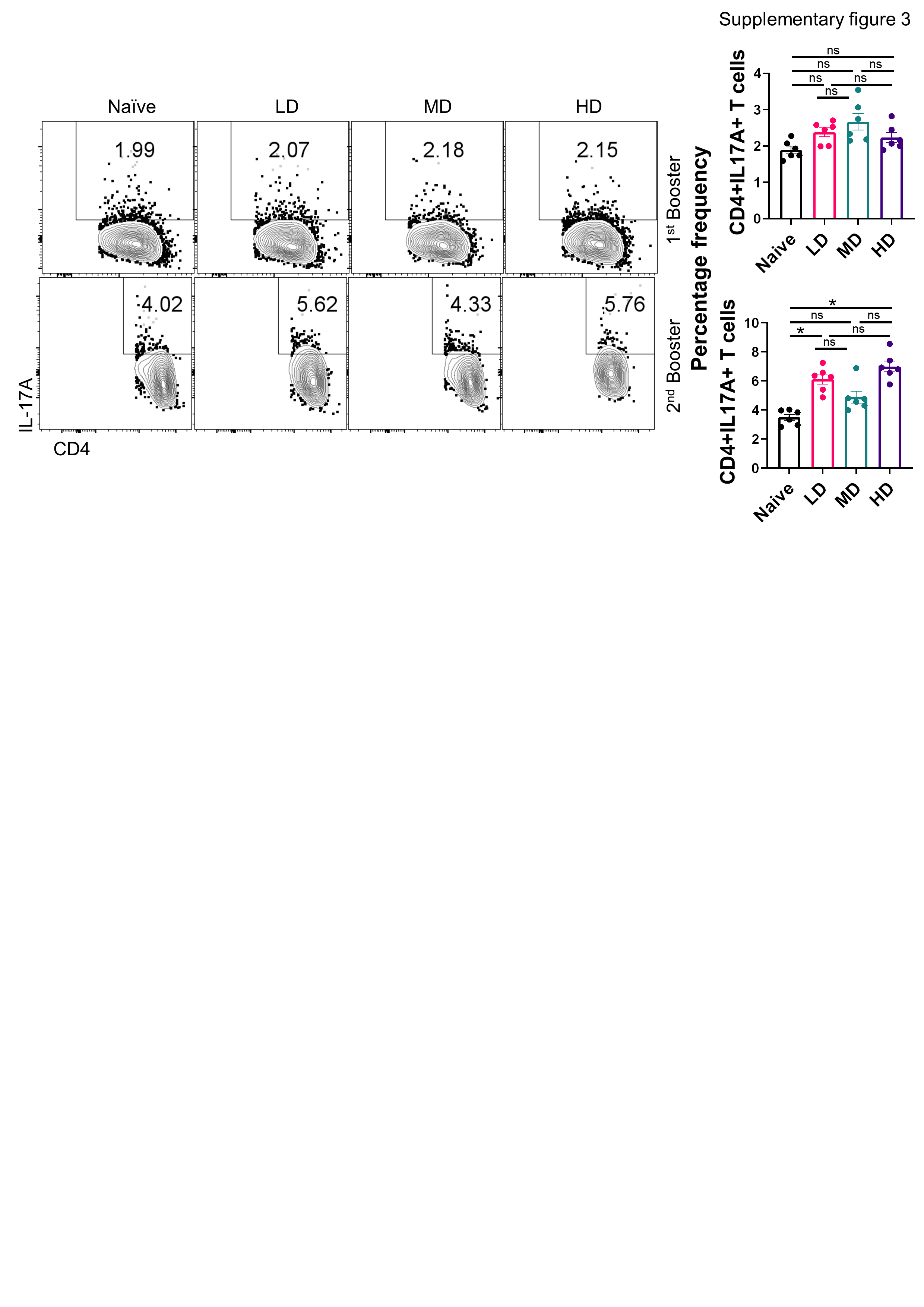

Supplement: Supplementary Figure 3 — Evaluation of Th17 cell response in CoviWall immunized C57BL/6 mice. Splenocytes from CoviWall immunized mice were used after 1st booster (top panel) and 2nd booster (lower panel) to evaluate Th17 response by evaluating CD4+IL17A+ T cell frequency by FACS. (left panel) Representative FACS contour plot and (right panel) bar graph showing mean ± SEM percentage frequency. For each experiment N=5. One way-Anova using non-parametric Kruskal-Wallis test for multiple comparison. *P < 0.05. [file Image3.tif]
